# A Mobile Phone App to Support Self-Management and Transition to Adult Health Services in Young People With a Chronic Illness: Single-Arm Pilot Intervention Study

**DOI:** 10.2196/67061

**Published:** 2025-09-03

**Authors:** Shehani C Samarasinghe, Riham Al Na'abi, Hoi Lun Cheng, Jeffrey Yeung, Katharine S Steinbeck

**Affiliations:** 1 Academic Department of Adolescent Medicine, The Children's Hospital at Westmead, Locked Bag 4001, Westmead, 2145, Australia, 61 2 78252148; 2 Speciality of Child and Adolescent Health, Faculty of Medicine and Health, The University of Sydney, Westmead, Australia

**Keywords:** adolescent, chronic illness, transition to adult care, self-management, mobile applications, pilot projects, diabetes mellitus, inflammatory bowel disease, cystic fibrosis, mobile phone

## Abstract

**Background:**

Pediatric to adult health service (AHS) transition is a challenging time for many adolescents and young adults with chronic illness. As the responsibility of illness management shifts from parents to the young person themselves, many young people fail to transition in a timely manner, which has important health consequences. Mobile apps show potential in assisting young people to self-manage their condition during this vulnerable time, but empirical data on app uptake as well as efficacy with respect to transition outcomes and illness control are lacking.

**Objective:**

This study aimed to evaluate the usage of a mobile app called *“*TransitionMate” (TMApp) by adolescents and young adults, which was purpose-built to support chronic illness self-management for youth undergoing transition. Secondary aims were to assess AHS attendance and changes in illness control over the first 12 months post transition. Upon demonstration of TMApp feasibility (49/70, ≥70% of participants using TMApp at least once a month), a randomized controlled trial was planned to test app efficacy in relation to transition and illness control outcomes.

**Methods:**

Young people aged 16‐19 years who were transitioning out of 2 major pediatric hospitals in Sydney, New South Wales, Australia, were recruited. Just before transition, participants’ demographic and clinical details were collected, and TMApp was installed on their mobile devices. Participants were contacted by telephone at 6 and 12 months for information on self-reported usage, app usefulness, and other feedback. Quantitative cohort-level usage data, including the number of times specific app features were accessed, were tracked via mobile analytics. At 12 months, electronic medical records from participants’ designated AHS were accessed for data on AHS attendances, measures of illness control, and unplanned hospitalizations.

**Results:**

In total, 73 adolescents and young adults (30 male; median age 18, IQR 17-18 y) were recruited, with 1 withdrawing participation in the first month. Participants were primarily from 3 chronic illness subgroups—diabetes mellitus (n=23), inflammatory bowel disease (n=19), and cystic fibrosis (n=9). Of the total, 50% (36/72) of participants reported using TMApp at 1 month post transition. Self-reported usage rates fell to 25% and 11% at 6 and 12 months, respectively. Mobile analytics data broadly corroborated self-reported usage. Furthermore, 8 participants who continued to use TMApp for 12 months gave a median usefulness rating of 8/10. Over two-thirds (48/72, 68%) of participants successfully transitioned to their designated AHS by 12 months. Among the successful transitioners who had illness control data available on electronic medical records, over 80% (24/30) maintained a stable or improved illness status at 12 months.

**Conclusions:**

TMApp was not used regularly enough by our young people to demonstrate feasibility and justify progression to a randomized controlled trial. Despite low app uptake, most participants successfully transitioned, suggesting that TMApp had minimal influence on transition outcomes.

## Introduction

Mobile phone technology is a significant part of everyday life and is seen as an attractive tool for both health care and health research. The management of chronic illness, where patients must maintain day-to-day routines to have good control of their condition, is a potential clinical situation where mobile apps might be useful.

One particular age group that may benefit from using mobile apps to manage their chronic condition is adolescents and young adults. First, young people, as digital natives, are confident users of mobile technology [1]. Second, adolescents undergo a challenging period of transition from pediatric to adult health services (AHS), whereby the responsibility of managing their chronic condition shifts substantially from the parent to the young person themselves [2]. Consequently, many young people neglect to monitor their health, adhere poorly to existing therapy, lose contact with any health care, and fail to transition in a timely manner [3,4]. Factors further contributing to suboptimal transition include inadequate communication between pediatric and adult services [5], decreased condition-specific illness knowledge in the AHS [6], and the lack of equivalent AHS [7]. Many of these issues are common among this age group, irrespective of chronic condition. Enhancing young people’s capacity to competently and autonomously self-manage their condition is thus a key strategy to retaining them in the AHS and improving their transition experience [8,9]. Importantly, self-management skills are imperative for preventing loss of illness control and unplanned hospitalizations arising from acute morbidity [10,11].

While it is often assumed that adolescents and young adults will embrace mobile apps for managing their health, evidence to support this assumption is modest. In total, 2 systematic reviews to date have assessed the use of apps for youth self-management of chronic illness [12,13]. One of these reviews focused on transition and found some improvement in patient satisfaction related to self-management following the use of apps [13]. However, neither study was able to draw conclusions about app efficacy pertaining to transition outcomes, owing to insufficient and heterogeneous data. Other studies, such as those evaluating the “iManage” app (Cincinnati Children’s Hospital Medical Center) for youth self-management of sickle cell disease [14-16], have generated useful information on app design, functionality, and acceptability with respect to consumer involvement, although efficacy data are yet to be available. Thus, knowledge gaps exist around understanding what role mobile apps play in the self-management of chronic illness for adolescents and young adults. Specifically, there is a need for more quantitative data on app usage and comparison of app uptake across different chronic conditions [17,18]. Empirical evidence on app efficacy with respect to transition outcomes and illness control is also lacking.

This study describes pilot testing of a mobile app called “TransitionMate” (TMApp), which was purpose-built to support adolescents and young adults in self-management during the vulnerable period of transition from pediatric to AHS. The app was designed as a generic (non–illness-specific) tool to address common issues around transition that could be used broadly across different health care settings and by patients with different conditions. The primary aim of this study was to evaluate young people’s usage of TMApp. Secondary objectives were to assess AHS attendance and changes in illness control over the first 12 months post transition. The working hypothesis was that regular and long-term usage of TMApp would predict successful transition. Upon demonstration of app feasibility (defined as a minimum usage frequency of once a month by ≥70% [49/70] of participants based on power calculations), a randomized controlled trial was planned to test efficacy of TMApp in relation to transition and illness control outcomes [19]. Both the primary and secondary objectives were necessary before embarking on the randomized controlled trial.

## Methods

### TMApp

TMApp was developed from a prototype that was designed in collaboration with the University of Sydney School of Electrical Engineering, involving a team of medical professionals and software engineers [20].

A brief iterative participatory design process was used for developing the prototype. Initial ideas around what functionalities to include were informed by patient needs identified in the transition literature [21-23], through clinician experience, as well as experience from the software engineers who had previously built mobile apps for adult chronic illness. Furthermore, a focus group involving 10 adolescents and young adults with chronic illness was conducted to gain further insights into what young people wanted from a self-management app. Five required features were identified in this process, including automated reminders, the ability to log and view trends for illness data, a medication list, a mood tracking function, and a centralized repository for storing important contacts, referral letters, and medical histories as young people often struggled to recall this information when speaking to a new health care provider [24].

A prototype was subsequently built using the above information. Screenshots of each feature were presented to several clinicians and researchers with expertise in adolescent health, and further refinements were made accordingly. This review-and-refine process was repeated several times. Following this, the prototype was tested in a group of young adults with chronic illness who had recently undergone transition [25]. Feedback received from this user group was the need for a to-do list as well as customizable content and displays to suit individual illness management needs, among other improvements (eg, graphics and bug fixes). This feedback, along with the prototype, provided the framework for a commercial developer to build TMApp in its final form. Overall, the commercial app development process was lengthy, interrupted by insufficient funds, and resulted in the relatively simple TMApp, that again had user testing with further refinements.

**Figure 1.**
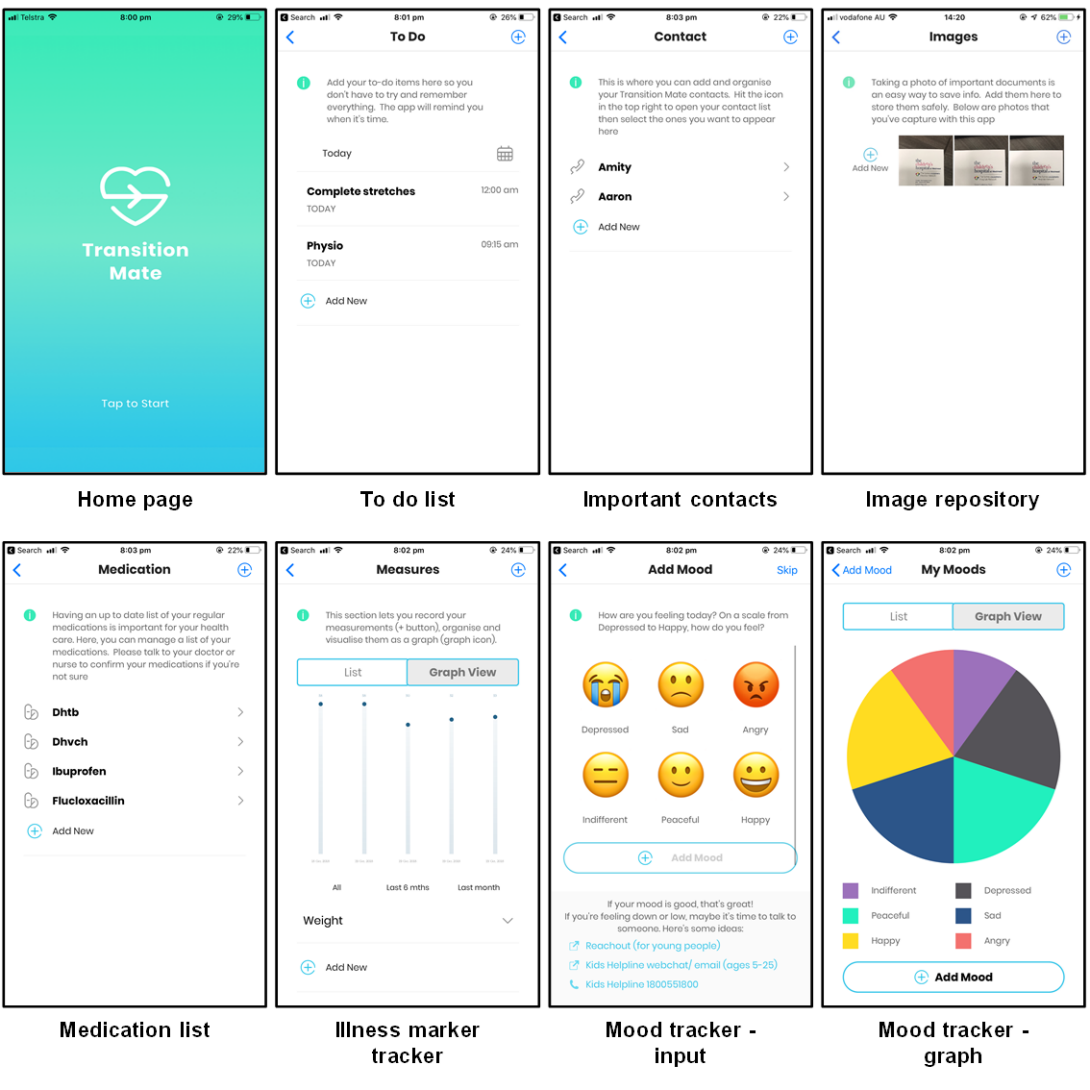
Screenshots of the main features on the TransitionMate mobile app.

The final version of TMApp had the following features: (1) to-do list; (2) reminders for health appointments and other health-related activities; (3) contact information for health care and emergency professionals; (4) image repository for prescriptions, reports, results, and clinic letters; (5) medication list; (6) tracking of illness control markers; and (7) an emoji-based mood tracker (Figure 1). Participants received monthly automated notifications via the app to encourage use. These notifications included prompts for setting goals, checking medications, and obtaining new prescriptions, with no reply option available.

TMApp was deployed on 2 major mobile operating systems—Apple iOS and Android (Google). For Apple iOS, TMApp was deployed through the App Store, whereby participants were given a promo code for free download onto their mobile device. To limit unintended downloads, it was made clear in the App Store that TMApp was intended for research participants only. For Android, deployment was through an Android Package Kit (.apk) file that was downloadable via a weblink emailed directly to participants. Technical support for TMApp was provided remotely by the commercial developer.

### Study Design and Setting

This pragmatic, single-arm, pilot trial was conducted at 2 tertiary pediatric hospitals in Sydney, New South Wales, Australia—The Children’s Hospital at Westmead and Sydney Children’s Hospital, Randwick.

The main study time points were baseline, 6 months, and 12 months. The baseline visit occurred during the final routine clinic appointment at the pediatric hospital and involved the collection of patient demographic, clinical, and contact details, including an assessment of psychological distress and transition readiness. Installation and setup of TMApp also occurred during this visit. Follow-ups at 6 and 12 months involved a telephone-administered semistructured questionnaire on (1) TMApp usage in the past month (daily, weekly, monthly, or never), (2) app features used most frequently and why, (3) AHS attendance (if they have attended or booked an appointment), and (4) illness control knowledge (if they could recall their markers of illness control and if any tests were done post transition). One additional question was included at 6 months, which asked participants to recall their TMApp usage during the first month of the trial. The 12-month questionnaire sought additional feedback, including a usefulness rating, whether TMApp was easy to use, whether they would recommend TMApp to others, and suggested improvements. Electronic medical record (eMR) data were sought from participants’ designated AHS at 12 months, including (1) the number of AHS attendances in the past year; (2) primary, and if available, secondary measures of illness control; and (3) unplanned hospitalizations in the previous 12 months. Further details on the methodology are provided in the full trial protocol [19]. Reporting of this research study is in accordance with the CONSORT (Consolidated Standards of Reporting Trials) Pilot Trials checklist (Checklist 1).

### Recruitment and Eligibility

Participants were recruited from May 2019 to May 2020 (between COVID-19 pandemic lockdowns). Adolescents with a chronic illness were approached with a study information pack by their treating teams during their penultimate routine clinic appointment. Patients were eligible if they were aged 16‐19 years, undergoing transition to an AHS within the next 12 months, and consented to using TMApp and being contacted for study follow-up. Patients were excluded if they were non–English speaking, had no access to a personal smartphone, had an intellectual disability affecting questionnaire completion, had a primary diagnosis of mental health, active cancer, or were in a terminal state, or were using another transition-focused mobile app.

### Transition Outcomes and Illness Control: Measurement and Definitions

#### Transition Outcomes

Using the eMR data obtained from AHS, we defined engagement as ≥1 recorded attendance at the participant’s designated AHS within the first 6 months post transition. Retention was defined as ≥1 further attendance between 6 and 12 months post transition. If AHS appointments were expected to be annual, 1 attendance within the 12-month study period was defined as engagement. A participant was deemed to have transitioned successfully if they had ≥1 AHS attendance by 12 months. Unsuccessful transitioners were defined as those who did not engage with the AHS they were referred to or could not be contacted to ascertain if they had transitioned elsewhere.

#### Illness Control

Quantitative measures of illness control (primary and, if available, secondary) were obtained at baseline by accessing eMR data from the pediatric health service. At the end of the study, participants’ designated AHS were contacted to obtain eMR data on subsequent measures of illness control and the number of unplanned hospitalizations.

Markers of illness control were specific to each chronic condition and were provided by clinicians from each pediatric specialty service, generally based on empirical evidence. Examples of common and clinically accepted markers of illness control are illustrated in Table 1. Participants were deemed to have experienced a deterioration in illness control if their primary or secondary markers showed a value that reflected a worse clinical state for their condition. Conversely, no change or a positive change in markers over time was indicative of stable or improved illness control.

**Table 1.** Clinically accepted markers of illness control and definitions for deterioration in illness control over 12 months.

Chronic illness and markers of illness control	Definitions for deterioration
Diabetes mellitus (type 1 and 2)	
HbA_1c_^a^	≥1% increase
Cystic fibrosis	
FEV1^b^	≥10% decrease
Weight^c^	≥5% decrease
Inflammatory bowel disease	
CRP^d^	≥75% increase from ULN^e^
Weight^c^	≥5% decrease
Other chronic illnesses	
Hospitalization	≥1 unplanned hospitalization related to chronic illness

a HbA_1c_: glycosylated hemoglobin.

b FEV1: forced expiratory volume (liters per second).

c Weight was considered a secondary illness marker for cystic fibrosis and inflammatory bowel disease.

d CRP: C-reactive protein.

e ULN: upper limit of normal (based on normative range defined by the laboratory providing the measure.

### Psychological Distress and Transition Readiness

Participants completed the Kessler Psychological Distress scale (K10) and the Transition Readiness Checklist (TRC) at baseline. The K10 is a validated, 10-item scale that assesses an individual’s mental distress related to depression and anxiety symptomatology [26]. The TRC is a 27-item tool developed by the Royal Children’s Hospital (Melbourne, Australia) that assesses self-management skills covering the areas of illness knowledge, administrative and organizational capabilities, and self-advocacy [27].

### Mobile Phone Analytics Data

Quantitative, cohort-level data on TMApp usage were obtained from Flurry Analytics (Flurry Inc). Variables of interest were the number of TMApp-active devices for each month of the study and the number of times each app feature was accessed. Due to privacy reasons, it was not possible to link mobile analytics data to individual user activity.

### Statistics

#### Power Calculation

An estimated 70 participants were needed to achieve a minimum sample size of 49, with attrition predicted at 30%. A sample of 49 participants would have ≥80% power, based on a 1-sided significance level of 0.05, to detect an increase in the rate of successful transition from pediatric to adult care by 15% (eg, from 70%‐85%). This sample size was considered feasible given the number of adolescents with chronic illness who transition out of the 2 study hospitals annually.

#### Data Analyses

Results are presented as data aggregated for the entire cohort, except where sex differences were considered and when condition numbers were large enough for subgroup analyses. Normality was assessed via the Kolmogorov-Smirnov test. Associations between categorical variables were analyzed using chi-square tests. Mann-Whitney *U* tests were used to compare transition outcomes between subgroups. Significance was set at *P*<.05. Summary statistics are presented as means (SD) or median (IQR) for continuous variables, and frequency or percentage for categorical variables. Statistical analyses were performed using IBM SPSS Statistics version 24 (IBM Corp).

### Ethical Considerations

The study was approved in 2017 by the Sydney Children’s Hospital Network Human Research Ethics Committee (HREC/17/SCHN/385). Due to lack of human resources and a prolonged app development process, the study did not commence recruitment until 2019. Signed informed consent was sought from parents and carers or from adolescents and young adults if they had reached 18 years.

Participants did not receive compensation for their involvement in the research.

All hard copy data (including questionnaire, phone call, and interview data) are stored in a locked filing cabinet in The Children’s Hospital at Westmead, Academic Department of Adolescent Medicine. Access to these data is restricted to staff involved in the research. Research data are deidentified upon receipt by the investigators using participant code numbers to preserve anonymity. Deidentified digital data are stored under password protection on a restricted-access network drive within the Sydney Children’s Hospital Network. Only the principal investigators have access to the participant reidentification list, and this list is stored separately as hard copy data. All data collected for the study will be preserved for 7 years from the date of obtaining maturity for young people who are older than 18 years, or for 7 years after the completion of the study for those aged more than 18 years. The hard and soft copies will be destroyed at this time under specific Human Research Ethics Committees protocols for destruction of paper and digital data.

## Results

### Participant Characteristics

**Figure 2.**
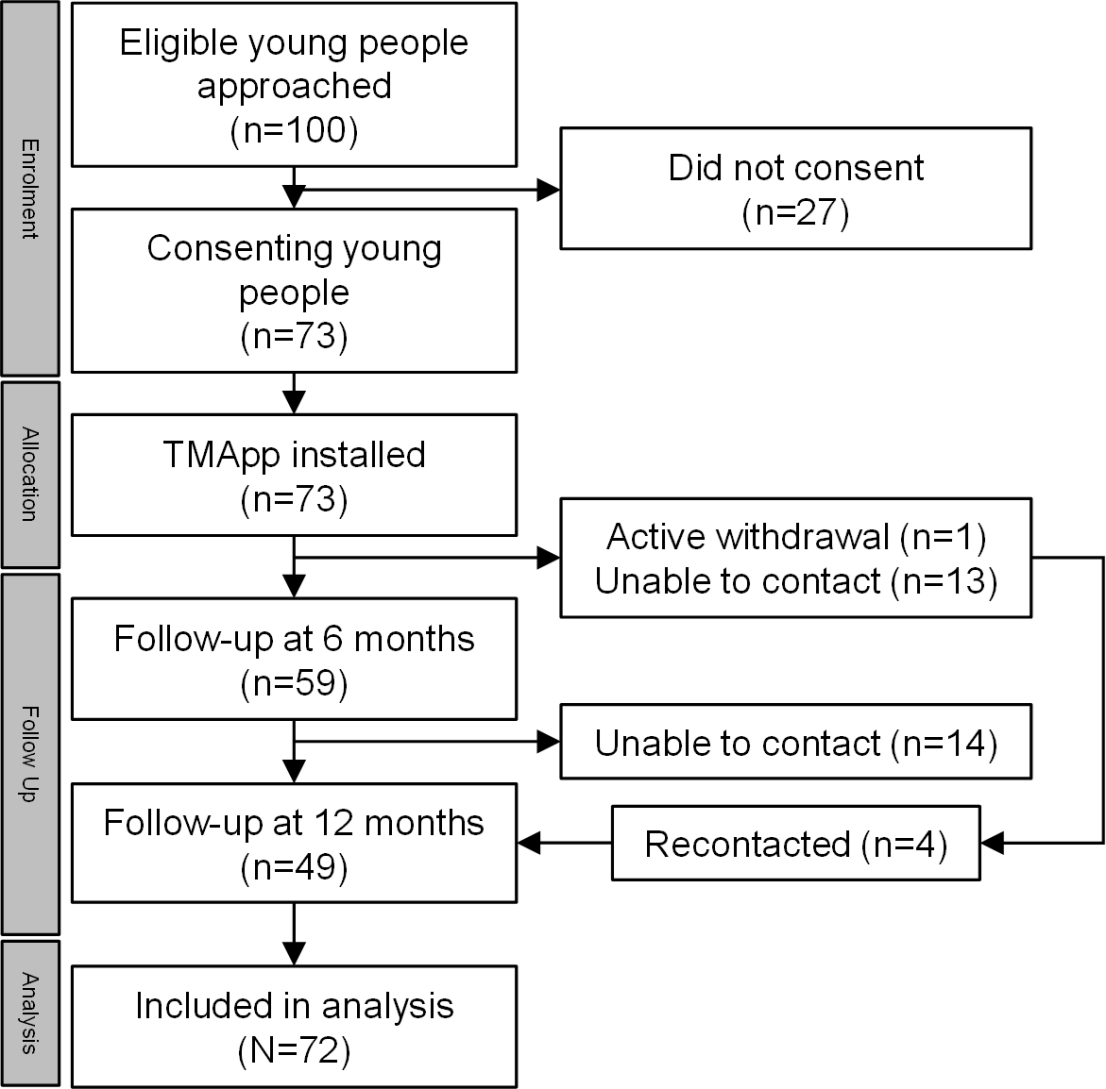
Study flowchart. Of the 73 participants who consented to this research, only 1 was excluded from analysis due to withdrawal of consent. TMApp: TransitionMate app.

A total of 100 adolescent patients were approached, with 73 (30 male; median age 18, IQR 17-18 y) consenting to take part in this study. One participant withdrew within the first month of the study, citing lack of interest and was excluded from analyses. At 6 and 12 months, 83% (60/72) and 68% (49/72) of participants could be contacted for follow-up, respectively (Figure 2).

The majority (51/72, 70%) of participants were from 3 chronic illness subgroups: diabetes mellitus (type 1 and 2; n=23), inflammatory bowel disease (n=19), and cystic fibrosis (n=9). Other chronic conditions in the remaining participants are outlined in Table 2. Median scores for the K10 (17 out of 50) and TRC (70 out of 81) were, respectively, in the lowest category for mental distress and the highest category for transition readiness (Table 2).

**Table 2.** Baseline participant characteristics. Data are presented as mean (SD) or median (IQR).

Participant characteristic	All (n=72)	Male (n=30)	Female (n=43)
Age (y), median (IQR)	18 (17‐18)	18 (17‐18)	18 (17‐18)
Chronic illness, n			
Type 1 diabetes mellitus	20	6	14
Type 2 diabetes mellitus	3	0	3
Inflammatory bowel disease	19	11	8
Cystic fibrosis	9	5	4
Other^a^	21	7	4
Kessler Psychological Distress Scale (K10)^b^^,c^, median (IQR)	17 (13‐24)	14 (12‐18)	20 (10‐42)
K10 response categories, n			
Likely to be well	43	22	21
Mild	12	3	9
Moderate	8	2	6
Severe	8	1	7
Transition Readiness Checklist (TRC)^c^^,d^	Median 70 (IQR 66‐76)	Mean 69 (SD 9)	Mean 69 (SD 8)
TRC categories, n			
Unlikely transition ready	4	2	2
Transition ready	67	26	41
Mobile operating system, n			
Android	53	23	30
Apple iOS	19	7	12

a Other conditions included: liver transplantation for congenital hepatic conditions (n=3); Duchenne muscular dystrophy (n=2); acromicric dysplasia (n=1); systemic lupus erythematosus (n=1); autoimmune thyroiditis (n=1); Grave’s disease (n=1); repaired duodenal atresia with consequent Barrett’s esophagus (n=1); bicuspid aortic valve with moderate aortic valve stenosis (n=1); neurofibromatosis type 1 (n=1); trichorhinophalangeal syndrome type 1 (n=1); epilepsy (n=1); glycogen storage disease pre-diabetes with severe metabolic syndrome (n=1); sinus node dysfunction with sinus arrest (n=1); constipation with fecal incontinence and megarectum (n=1); idiopathic megarectum (n=1); acquired brain injury (n=1); hereditary spastic paraplegia (n=1); undefined neurodegenerative disorder (n=1).

b Categories for K10 scores: Likely to be well (<20), mild mental disorder (20-24); moderate mental disorder (25-29), severe mental disorder (≥30).

c One participant had missing data for the K10 and TRC.

d Categories for TRC scores: Unlikely transition ready (27-54); Transition ready (55-81).

### TMApp Usage

**Figure 3.**
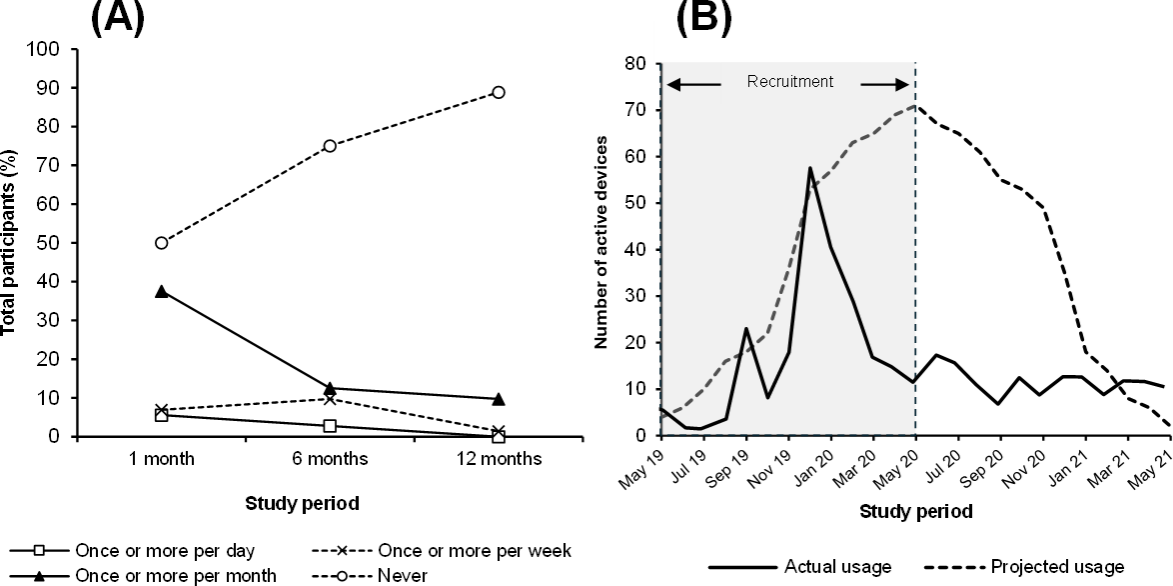
TransitionMate app usage data based on (A) self-reported frequency in the past month; and (B) mobile analytics data (black line) mapped against projected usage if every participant accessed the app on their device every month. (N.B. actual usage was higher than projected usage at some time points as mobile analytics data include user activity on researcher-held devices).

At 6 months, 36 of the 72 (50%) participants reported having used TMApp at least once in the first month post transition, with 4 daily and 5 weekly users (Figure 3). By 6 months, past-month TMApp usage decreased to 18 (25%) participants, with 2 daily and 7 weekly users. At 12 months, 8 (11%) participants reported past-month TMApp usage, with no daily and 1 weekly user.

Aggregated usage data from mobile analytics showed peak usage at mid-recruitment, after which TMApp usage rates fell well below projected rates calculated based on monthly use by all participants (Figure 3). At study completion, TMApp was removed from the iOS App Store to limit unintended downloads and contamination of app usage statistics.

**Figure 4.**
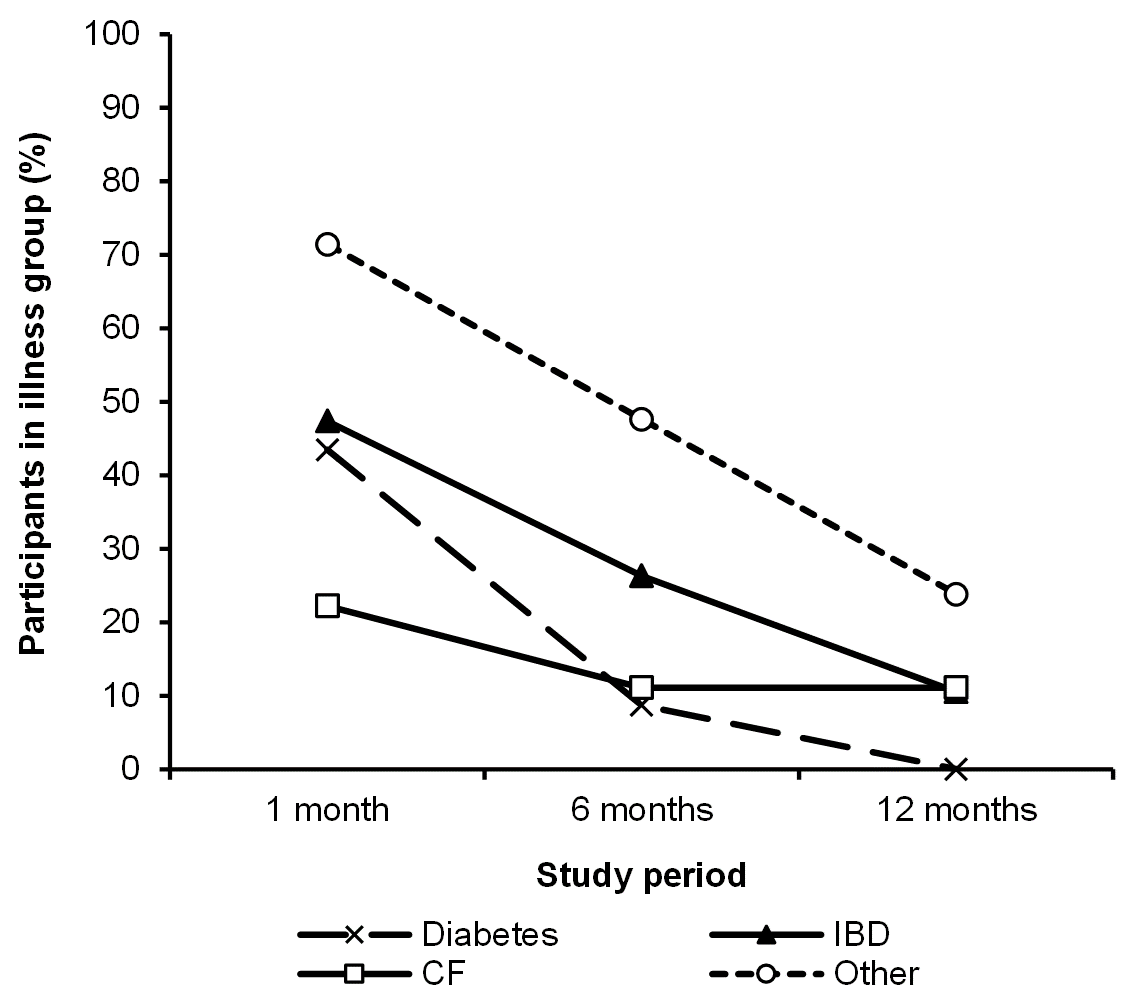
Proportion of participants within each illness subgroup who reported using the TransitionMate app at least once over the past month. CF: cystic fibrosis; IBD: inflammatory bowel disease.

Across the chronic illness subgroups, self-reported TMApp usage was highest among participants in the “other” group (Figure 4). Diabetes, being the largest chronic illness subgroup, had the second lowest self-reported TMApp usage at 1 month (10/23, 43% reported use) and the lowest TMApp usage at 6 months (2/23, 9%). No participants with diabetes reported using the TMApp at 12 months.

**Figure 5.**
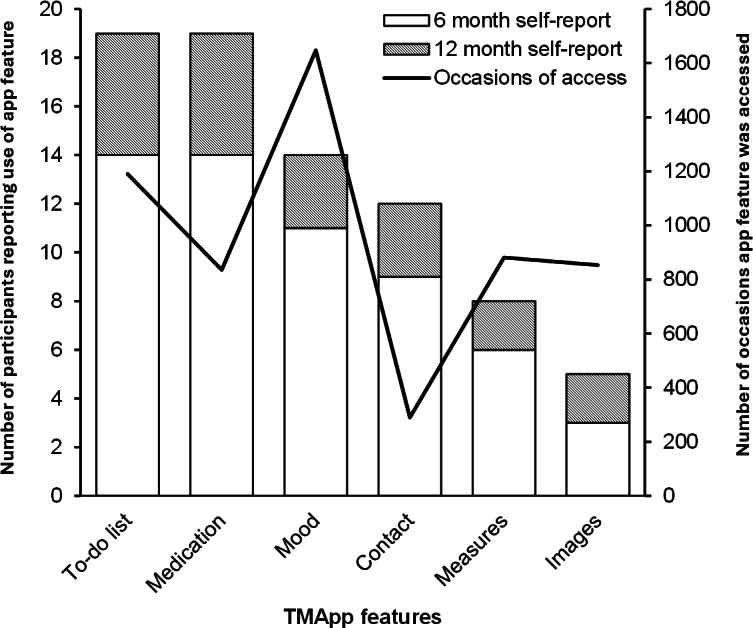
Use of TransitionMate app features based on: self-report at 6 months (white bars), 12 months (hashed bars), and quantitative mobile analytics data (line). TMApp: TransitionMate app.

The top 3 most used features reported by participants were the to-do list (n=14), medication (n=14), and mood tracker (n=11) functions. Mobile analytics data broadly corroborated participant self-reports, with the mood tracker recorded as the most accessed feature over 12 months, followed by the to-do list and medication functions (Figure 5).

### TMApp Usefulness and Other Feedback

The primary reason participants gave for not using TMApp was that they felt no need to use it (n=24). Other reasons were forgetting to use the app (n=5), changing phones (n=4), low app usefulness (n=3), lack of time (n=1), and preferring paper to apps (n=1). In terms of potential improvements, participants suggested the incorporation of health records, nutritional information, additional visual components, and a medication reminder alarm feature. Furthermore, 7 participants stated that the existing features were adequate. Among the 8 participants who continued to use TMApp at 12 months, a median usefulness rating of 8 out of 10 was given. In addition, 16 participants found TMApp easy to use and would recommend it to other young people with a chronic illness as they transition into adulthood.

### 6- and 12-Month Outcomes

The eMR data obtained from AHS showed that 49 out of the 72 participants successfully engaged with their designated AHS by 6 months. By 12 months, 48 of these participants were retained in the same AHS. Males exhibited a nonsignificant tendency for higher AHS engagement (23/29, 79% vs 26/43, 60%; *P=*.09) and retention (23/29, 79% vs 25/43, 58%; *P=*.06) compared with females. The proportion of successful (n=49) versus unsuccessful (n=23) transitioners did not differ between chronic illness subgroups (diabetes: 17/23, 74%; inflammatory bowel disease: 13/19, 68%; cystic fibrosis: 8/9, 89%; other: 11/21, 52%; *P=*.21). Similarly, K10 and TRC scores as well as primary markers of illness control at baseline did not differ between the successful and unsuccessful transitioners (Table 3).

Markers of illness control (obtained from AHS eMR) for the 3 major illness subgroups remained stable over the course of the study (Tables 4-6). Around 80% of participants (for whom follow-up eMRs were available) showed stable or improved illness status at 6 months (17/21) and 12 months (24/30). In total, 8 participants had unplanned hospitalizations recorded on eMR, including 5 with cystic fibrosis, 2 with diabetes, and 1 with inflammatory bowel disease. Furthermore, 1 participant with cystic fibrosis had 3 hospitalizations over the study period.

Of the 23 participants who had not engaged with their designated AHS by 12 months, 17 were contactable at follow-up. Reasons given for nonattendance were: (1) participant had not yet chosen an AHS to visit (n=3), (2) parents were arranging appointments for a later date (n=2), (3) delaying transition until after their final school year examinations (n=2), (4) planning later transition (n=2), and (5) hesitancy over visiting a health service during the COVID-19 pandemic (n=2). Furthermore, 1 participant was uncertain how to schedule an appointment, and 5 did not offer an explanation.

**Table 3.** Comparison of baseline psychological distress and transition readiness scores and baseline markers of illness control between successful and unsuccessful transitioners. Data are presented as median (IQR). Statistical comparisons were performed using the Mann-Whitney *U* test.

Baseline measure	Successful transitioners^a^ (n=49)		Unsuccessful transitioners^a^ (n=23)		*P* value
	n	Median (IQR)	n	Median (IQR)	
Kessler Psychological Distress Scale (K10)^b^	48	16 (12‐21)	23	21 (13‐28)	.10
Transition Readiness Checklist (TRC)^c^	48	69 (66‐76)	23	71 (64‐76)	.60
Primary marker for illness group					
Diabetes mellitus (HbA_1c_^d^; %)	17	7.2 (6.9‐8.6)	6	7.7 (6.7‐9.3)	.92
Cystic fibrosis (FEV1^e^; L/s)	8	2.9 (2.3‐3.4)	1	3.4 (3.4-3.4)	—^f^
Inflammatory bowel disease (CRP^g^; mg/L)	12	1.2 (0.5‐2.2)	6	1.0 (0.8‐4.5)	>.99

a Successful transitioners were those who had ≥1 adult health service attendances on their electronic medical record at 12 months. Unsuccessful transitioners were those who did not have any recorded attendances at their designated adult health service at 12 months.

b Categories for K10 scores: Likely to be well (<20), mild mental disorder (20-24); moderate mental disorder (25-29), severe mental disorder (≥30).

c Categories for TRC scores: Unlikely transition ready (27-54); Transition ready (55-81).

d HbA_1c_: glycosylated hemoglobin.

e FEV1: forced expiratory volume.

f Not applicable as there was only 1 person in this subgroup.

g CRP: C-reactive protein.

**Table 4.** Change in illness control for the diabetes subgroup based on eMR^a^ data.

Diabetes mellitus	Primary illness marker	
	n	HbA_1c_^b^ (%), median (IQR)
Baseline	23	7.3 (6.9-8.6)
6 months^c^	10	8.3 (7.4-9.7)
Stable/improved, n	7	
Deteriorated, n	3	
12 months^c^	13	7.1 (6.8-8.9)
Stable/improved, n	10	
Deteriorated, n	3	

a eMR: electronic medical record.

b HbA1c: glycosylated hemoglobin.

c eMR records were only available for 37 participants due to limited data availability. A shift toward telehealth consultation in response to COVID-19 social distancing regulations wa as major contributor to missing data as many illness control assessments were not conducted.

**Table 5.** Change in illness control for the cystic fibrosis subgroup based on eMR^a^ data.

Cystic fibrosis	Primary illness marker		Secondary illness marker	
	n	FEV1^b^ (L/s), median (IQR)	n	Weight (kg), median (IQR)
Baseline	9	2.9 (2.3-3.4)	9	58.8 (52.5-62.8)
6 months^c^	1	2.3 (2.3-2.3)	1	59.2 (59.2-59.2)
Stable/improved, n	1		1	
Deteriorated, n	0		0	
12 months^c^	8	3.2 (2.1-3.6)	8	59.1 (53.5-65.4)
Stable/improved, n	5		6	
Deteriorated, n	3		2	

a eMR: electronic medical record.

b FEV1: forced expiratory volume (liters per second).

c eMR records were only available for 37 participants due to limited data availability. A shift toward telehealth consultation in response to COVID-19 social distancing regulations wa as major contributor to missing data as many illness control assessments were not conducted.

**Table 6.** Change in illness control for the inflammatory bowel disease subgroup based on eMR data.

Inflammatory bowel disease	Primary illness marker		Secondary illness marker	
	n	CRP^b^ (mg/L), median (IQR)	n	Weight (kg), median (IQR)
Baseline	19	1.0 (0.6-2.1)	19	62.4 (55.1-74.7)
6 months^c^	10	1.4 (0.4-7.7)	10	64.5 (52.8-93.8)
Stable/improved (n)	9		9	
Deteriorated (n)	1		1	
12 months^c^	9	3.9 (1.0-4.0)	9	69.0 (55.5-83.0)
Stable/improved (n)	7		8	
Deteriorated (n)	1		1	

a eMR: electronic medical record.

b CRP: C-reactive protein (the most widely used serum indicator of inflammation).

c eMR records were only available for 37 participants due to limited data availability. A shift toward telehealth consultation in response to COVID-19 social distancing regulations was major contributor to missing data as many illness control assessments were not conducted.

## Discussion

### Principal Findings

To our knowledge, this is the first clinical trial to evaluate the uptake of a generic app to enhance pediatric to AHS transition in young people living with a chronic illness. This was designed as a feasibility study to determine usage over the vulnerable 12 months after young people leave wrap-around pediatric care and have more autonomy. Results from both the self-reported and quantitative usage data showed that TMApp was not used frequently enough to justify feasibility and efficacy testing in a randomized controlled trial. Not needing to use the app and forgetting to use the app were the top reasons given for nonuse. Other contributors to low usage may include the generic (ie, non–illness specific) design of the app, transition perceived as being of low priority, and TMApp’s simple design compared with other commercial apps for managing chronic illness. The high usefulness ratings obtained from this study were likely to be biased, as feedback was only given by a small subset of highly engaged study completers. The nonanonymized nature of the follow-up interviews may have also yielded more positive but less truthful feedback. Given that we were unable to link the mobile analytics data to individual user activity, this study serves more as an observational study of the transition from pediatric to AHS.

TMApp was developed with input from adolescents and young adults to support transition in young patients with chronic illness. While the overall frequency of app usage was low with declining rates over time, TMApp was rated as useful and easy to use among those who did regularly access it to support their health service transition. This result is comparable with a pilot study of the “iManage” app for youth self-management of sickle cell disease. The iManage studies described challenges in maintaining user engagement despite observing improvements in self-efficacy and management of pain and mood [15]. Our attrition rates are also comparable with other studies of mobile health app use among adolescents and young adults [28,29]. Research suggests that enhancing brightness, color schemes, and gamification functions could boost health app usage among youth [30-32]. Other modifications, such as conversational style interactions, speech bubbles, avatars, and illness-specific customizations (eg, importing of glucose monitor data), that were suggested by young adults during prototype testing could have also boosted usage rates in this study, although budgetary constraints precluded their inclusion in the final version of TMApp. Adding social interaction elements (either with the health care team or peers with similar conditions) was also suggested by young people who used the app. Such features may improve engagement and help strengthen social support systems for young patients [33,34], although these would introduce safeguarding and privacy issues. A full-time app moderator would have been required to manage the online interactions and respond promptly to any health emergencies that could have been communicated through the app. Our research team had neither the human nor the financial resources to support this. Finally, while our app development process incorporated some aspects of participatory design, a greater level of youth collaboration using youth-led and co-design principles would have allowed us to better understand their self-management needs, motivations for health technology use, and could have resulted in better usage rates.

Over two-thirds of the cohort (49/72, 68%) successfully transitioned to their designated AHS by 12 months. By comparison, varying rates of successful transition have been described in other studies, which range from approximately 20% to 90% [35-38]. Neither sex, chronic illness subgroup, nor baseline levels of distress and transition readiness were significantly predictive of successful transition in our small cohort, despite some research suggesting that AHS engagement is higher among young females [39-41]. Our relatively high rate of successful transition is likely attributed to the efficient local clinical protocols governing patient transition to adult care [42,43], especially for the main illness subgroups. Ongoing parental assistance in scheduling AHS appointments, as alluded to by some participants, also likely facilitated successful transition. Our current data suggest that implementation of the TMApp in conjunction with existing clinical procedures would have made minimal contributions to improving the transition process. For the 6 participants who did not engage with their designated AHS and were uncontactable at follow-up, it was not possible to ascertain whether they failed to transition or sought care from a different AHS, and this population requires further study. A unique barrier to transition experienced in this study was anxiety around attending medical appointments during the COVID-19 pandemic. The shift toward telehealth during pandemic lockdowns may have also delayed transition plans for some participants, given that many young people express a preference toward face-to-face over telehealth consultations [44,45].

The majority of participants who engaged with an AHS showed stable or improved illness markers and had low hospitalizations (except for one participant with cystic fibrosis). While we were unable to track illness control and hospitalizations in those who did not attend their designated AHS, our data support proactive follow-up for no-shows at AHS.

The key strengths of the study are, first, the follow-up methodology that involved obtaining posttransition eMR data on health service engagement and illness control, thus reducing reporter burden and error. Second, the mobile analytics data served as a valuable tool to quantify true app usage, despite only aggregate data being available due to user privacy restrictions. Third, TMApp was a generic mobile app designed to cover a wide range of chronic illnesses. This improves equity of access for individuals with rarer conditions where illness-specific self-management apps may not be available, but also limits the extent to which the app could be personalized. The major limitation was undoubtedly the low rates of usage, and the reasons for this have been discussed. Outsourcing app development to a commercial entity also introduced financial and logistical constraints that resulted in a relatively rudimentary TMApp design. Based on our current experiences, in-house development and maintenance are the only viable options for mobile health apps that have a small user base.

### Conclusions

This pilot study did not demonstrate the feasibility of TMApp as it was not used regularly enough by adolescents and young adults with chronic illness in the first 12 months of transition. Over two-thirds of participants successfully transitioned despite low app usage, which is likely attributed to effective transition care protocols already in place, among other facilitators. Enhancing features, such as gamification and social networking capabilities, could help increase young people’s app uptake and need to be tested. Importantly, future studies of mobile health apps for adolescents and young adults should include objective usage metrics like mobile analytics, as standard user feedback can be subject to considerable bias.

## Supplementary material

10.2196/67061 Checklist 1 CONSORT Pilot Trials checklist.

## ABBREVIATIONS

AHS: adult health service
CONSORT: Consolidated Standards of Reporting Trials
eMR: electronic medical record
K10: Kessler Psychological Distress Scale
TMApp: TransitionMate
TRC: Transition Readiness Checklist
